# Principles and Illustrations of Morbid Anatomy

**Published:** 1834-01-01

**Authors:** 


*Principles and Illustrations of Morbid Anatomy.* By J. HOPE, M.D., F.R.S., Physician to the St. Mary-le-bone Infirmary. Part VII.—London, 1833. 8vo.

THE subject of the present part is Ulceration of the Alimentary Canal, of which a very fair account is given. In this, as in many other instances, we quote only a small part of the title of the book, as Dr. Hope, following many modern but bad examples, squeezes a whole preface into a title-page. The plates, four in number, are remarkably well executed.

